# Couples with mild male factor infertility and at least 3 failed previous IVF attempts may benefit from laparoscopic investigation regarding assisted reproduction outcome

**DOI:** 10.1038/s41598-020-59170-5

**Published:** 2020-02-11

**Authors:** Agni Pantou, Konstantinos Sfakianoudis, Evangelos Maziotis, Polina Giannelou, Sokratis Grigoriadis, Petroula Tsioulou, Georgia Kokkali, Michael Koutsilieris, Konstantinos Pantos, Mara Simopoulou

**Affiliations:** 1 Centre for Human Reproduction, Genesis Athens Clinic, Athens, Greece; 2 Department of Physiology, Medical School, National and Kapodistrian University of Athens, Athens, Greece; 3 Assisted Conception Unit, 2nd Department of Obstetrics and Gynaecology, Aretaieion Hospital, Medical School, National and Kapodistrian University of Athens, Athens, Greece

**Keywords:** Reproductive techniques, Infertility

## Abstract

The aim of this study is to assess the value of laparoscopy for couples diagnosed with mild male factor infertility and at least three previous failed *In-Vitro* Fertilization (IVF) attempts. A total of 169 couples were included in this prospective cohort study. Patients were presented with the option of being subjected to laparoscopic investigation for correction of previously unidentified endometriosis or pelvic adhesions. The outcome measures were Live Birth/Ongoing Pregnancy, clinical pregnancy and positive hCG rate. One-hundred and one of them opted for, whereas 68 opted against laparoscopic investigation. All patients proceeded with a single ICSI cycle. Following laparoscopic investigation, 43 patients were diagnosed with endometriosis, 22 with adhesions, while for 36 patients laparoscopic investigation provided no further diagnosis. No statistically significant differences were observed regarding baseline hormonal levels and other characteristics between the two groups and the three subgroups. When compared to the no-laparoscopy group, women subjected to laparoscopy presented with a higher clinical pregnancy and ongoing pregnancy/live birth rate. Following endometriosis correction, a marginally non-statistically significant trend was observed regarding a decrease in poor-quality blastocysts (p = 0.056). A statistically significant higher clinical pregnancy (p = 0.03) and ongoing pregnancy/live birth rate was observed in the endometriosis group when compared to male factor infertility only (p = 0.04). Laparoscopic identification and correction of undiagnosed endometriosis in couples initially diagnosed with male infertility and at least 3 failed previous IVF attempts, appears to be a promising approach efficiently addressing infertility for these patients while avoiding IVF overuse.

## Introduction

Male factor infertility stands as a common infertility etiology, identified in up to 50% of infertile couples^1^, as the sole etiology or coupled with female factor infertility. Studies on male factor infertility, provide robust data that this may be successfully managed through in vitro fertilization (IVF) treatment, ensuring high implantation and pregnancy rates^2^. In fact, even severe and challenging cases of male factor infertility could be effectively managed, through the employment of a wide pallet of techniques improving standard employment of Intracytoplasmic Sperm Injection (ICSI) along with fertilization rates^3^. Male infertility has been categorized as mild, moderate or severe^4^. Mild male factor infertility does not present with a strict definition, including a range of different pathologies. It is defined by the observation of a single abnormal finding of the semen analysis or by a total motile sperm count between 10–20 × 10^6^/mL^5,6^. Couples with mild male factor infertility, according to guidelines, should proceed with IVF, and not Intrauterine Infusion (IUI), following 24 months of failed attempts to conceive naturally (NICE Guidance. For people with unexplained infertility, mild endometriosis or mild male factor infertility, who are having regular unprotected sexual intercourse do not routinely offer intrauterine insemination, either with or without ovarian stimulation (exceptional circumstances include, for example, when people have social, cultural or religious objections to IVF) advise them to try to conceive for a total of 2 years (this can include up to 1 year before their fertility investigations) before IVF will be considered. (NICE https://www.nice.org.uk/donotdo/for-people-with-unexplained-infertility-mild-endometriosis-or-mild-male-factor-infertility-who-are-having-regular-unprotected-sexualintercourse-do-not-routinely-offer-intrauterine-insemination-either-with-or-without-ovarian-stimulation-exceptional-circum). Mild factor infertility is regarded as a good prognosis regarding the outcome of an IVF/ICSI cycle, resulting to a relatively high clinical pregnancy rate following embryo transfer^7^.

A high number of previous failed IVF attempts affects a remarkable 30% of infertile couples^8^. This may be attributed to a wide range of infertility factors reported, such as severe male infertility^9^, autoantibodies^10^, as well as undetected genetic defects, or endometriosis in line with various uterine pathologies^11,12^, and unexplained infertility.

The driver behind this study was the authors’ aspiration to investigate why couples with a good prognosis of mild male factor and no female infertility indication may subjected-perhaps inexplicably-to more than 3 failed IVF attempts. This prospectively designed study focuses on whether couples with mild male factor infertility and more than 3 failed IVF cycles could benefit from further diagnostic examination namely, performing diagnostic and corrective laparoscopy as the end-point of infertility investigation. The aim of this study is to evaluate whether such an approach could address and manage successfully these cases, by allowing a successful single postoperative IVF treatment.

## Results

A total of 169 couples diagnosed with male factor infertility and a reproductive history of at least 3 failed IVF attempts were eligible to participate in the study. Sixty-eight of them opted not to be subjected to laparoscopy and proceeded with an ICSI cycle, while the remaining 101 opted for laparoscopy investigation. Out of the 101 women subjected to laparoscopy, 43 were diagnosed with endometriosis (42.57%) and 22 with pelvic adhesions (21.78%). For the remaining 36 patients no diagnosis of endometriosis was established following laparoscopy, hence this group is herein described as “male factor infertility only”. The findings of laparoscopy are presented in Supplementary Table 1.

**Figure 1:**
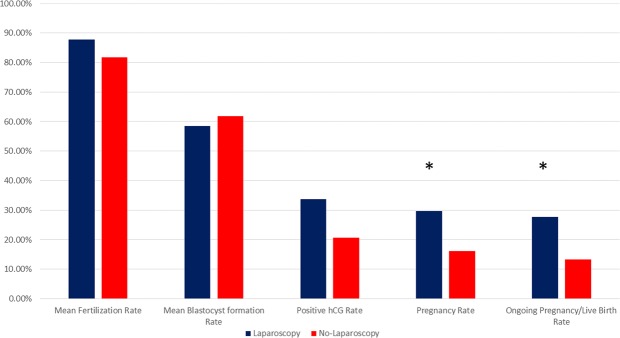
Mean fertilization, blastocyst formation, positive hCG, clinical pregnancy and live-birth rates for the laparoscopy and no-laparoscopy group respectively.

Age, previous IVF attempts, LH, FSH, E2 and progesterone levels did not differ statistically significantly between the patients that were submitted to laparoscopy and to those that opted against (Table 1). Patients that opted not to be subjected to laparoscopy presented with statistically significant less years of infertility compared to the laparoscopy group (4.68 ± 0.81 vs 7.28 ± 0.92, p < 0.001). Thirty-four out of 101 women that were submitted to laparoscopic investigation and 13 out of 68 women who opted against laparoscopy, presented with a positive hCG test (OR: 1.95; 95% CI: 0.95–4.01; p = 0.06). The clinical pregnancy rate in the laparoscopy group (30/101; 29,7%) was statistically significantly higher compared to the no-laparoscopy group (11/68; 16.18%) (OR: 2.19; 95% CI: 1.01–4.75; p = 0.04). Two miscarriages were reported for each of the groups resulting to 26 live-births and 2 ongoing pregnancies in the laparoscopy group, and 9 live births in the no-laparoscopy group. The laparoscopy group presented with a statistically significant higher ongoing pregnancy/live birth rate compared to the no-laparoscopy group (OR: 2.51; 95% CI: 1.10–5.74; p = 0.03) (Fig. 1).

**Table 1:** Descriptive statistics of the laparoscopy and no-laparoscopy groups, and the three laparoscopy subgroups.

	Laparoscopic investigation	No Laparoscopy	Endometriosis	Pelvic Adhesions	Male Factor Infertility only
Age	36.30 ± 1.42	36.09 ± 1.72	36.51 ± 1.26	36.00 ± 1.62	36.22 ± 1.42
Years of Infertility	7.28 ± 0.92	4.68 ± 0.81^a^	7.21 ± 0.93	7.14 ± 0.76	7.44 ± 0.98
Previous IVF attempts	4.32 ± 0.76	4.18 ± 0.64	4.47 ± 0.82	4.27 ± 0.86	4.19 ± 0.57
FSH (U/ml)	4.31 ± 0.71	4.26 ± 0.97	4.29 ± 0.65	4.42 ± 0.71	4.26 ± 0.78
LH (U/ml)	3.76 ± 0.88	4.03 ± 0.83	3.77 ± 0.87	3.72 ± 0.97	3.77 ± 0.82
Estradiol (pg/ml)	2855.51 ± 147.02	2874.82 ± 216.71	2888.35 ± 153.28	2830.23 ± 141.53	2831.72 ± 134.52
Progesterone (ng/ml)	12.86 ± 1.73	12.65 ± 1.69	12.64 ± 1.78	12.49 ± 2.05	13.33 ± 1.30
Oocytes Retrieved	10.82 ± 1.95	11.01 ± 1.78	11.30 ± 2.19	10.45 ± 1.75	10.47 ± 1.59
MII oocytes	9.37 ± 1.84	9.84 ± 1.8	9.65 ± 2.22	9.64 ± 1.61	8.86 ± 1.29
Fertilized oocytes	8.20 ± 1.53	7.84 ± 0.98	8.56 ± 1.82	8.41 ± 1.23	7.64 ± 1.08
Blastocysts	4.78 ± 0.95	4.78 ± 0.76	5.00 ± 1.08	4.82 ± 0.83	4.50 ± 0.76
Positive hCG test (%)	34/101 (33.66%)	14/68 (20.58%)	17/43 (39.53%)	8/22 (36.36%)	9/36 (25.00%)
Clinical Pregnancy (%)	30/101 (29.70%)^b^	11/68 (16.18%)	17/43 (39.53%)^c^	7/22 (31.82%)	6/36 (16.67%)
Live Birth (%)	28/101 (27.72%)	9/68 (13.24%)	16/43 (37.21%)^c^	6/22 (27.27%)	6/36(16.67%)

^a^statistically significant different when compared to no-laparoscopy group (p < 0.001), ^b^statistically significant different when compared to no-laparoscopy group, ^c^statistically significantly different when compared to male factor infertility only.

**Figure 2:**
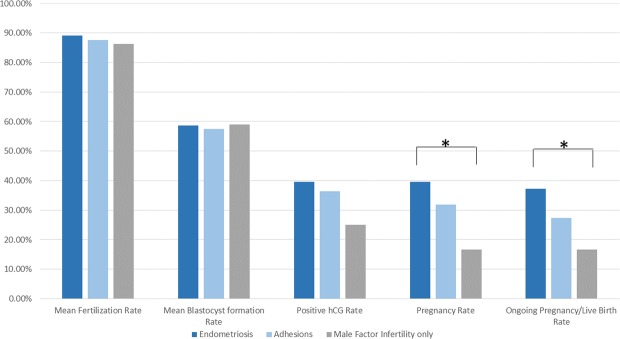
Mean fertilization, blastocyst formation, positive hCG, clinical pregnancy and live-birth rates for the endometriosis, adhesions and male factor infertility only subgroups of the laparoscopy group.

Age, previous IVF attempts, years of infertility, LH, FSH, E2 and progesterone levels did not differ statistically significantly between the three groups of patients that underwent laparoscopic investigation (Table 1). Following laparoscopy all patients proceeded with a single ICSI cycle. No statistically significant difference was observed between the two and the three subgroups regarding the number of oocytes retrieved, number of MII oocytes, number of normally fertilized oocytes and number of blastocysts (Table 1). Two blastocysts of the highest grading were transferred for each patient. Quality of the embryos transferred in previous cycles and in the present cycle is reported in Table 2. For the endometriosis group following correction, a trend was observed regarding a higher number of moderate embryo quality cycles when compared to poor embryo quality cycles, though marginally not statistically significant (p = 0.056). No other statistically significant difference was observed. No statistically significant difference was observed regarding the positive hCG rate between the three groups. The endometriosis group following correction presented with a statistically significant higher clinical pregnancy rate (OR: 3.27; 95%CI: 1.12–9.52; p = 0.03) and ongoing pregnancy/live birth rate (OR: 2.96; 95%CI: 1.01–8.66; p = 0.047) compared to the group classified as male factor infertility only group following laparoscopy No statistically significant difference on clinical pregnancy was observed regarding the pelvic adhesions group in comparison to any of the other groups (Fig. 2).

**Table 2:** Quality of embryos transferred in the pre and post recruitment study cycles.

	Embryo’s quality	No laparoscopy	Laparoscopy	Endometriosis	Pelvic Adhesions	Male Factor only
Pre-recruitment	Top	14	21	7	6	8
	Moderate	35	45	18	12	15
	Poor	19	35	18	4	13
Post-recruitment	Top	13 (6)	20 (9)	7 (4)	5 (2)	8 (3)
	Moderate	35 (5)	53 (18)	26 (11)	13 (5)	14 (2)
	Poor	20 (1)	28(3)	10 (2)	4 (0)	14 (1)

Number in brackets in the post-operative cycle represent the number of patients who achieved clinical pregnancy.

The mean age of our patients diagnosed with endometriosis was 36.51 (±1.26) years old. The number of previous failed IVF attempts ranged from 3 to 6 with an average of 4.47 (±0.82) previous failed IVF attempts. The years of infertility the patients reported ranged from 5 to 9 with an average of 7.21 (±0.93) years. Fourteen patients were diagnosed with stage I endometriosis, 25 with stage II, 4 with stage III and none with stage IV endometriosis. Seventeen out of the 43 patients presented with a positive hCG test, leading to a clinical pregnancy following a single ICSI cycle. No statistically significant difference was observed between the pregnant and the non-pregnant group for all parameters studied (Supplementary Table 2). Only one miscarriage has been reported for this subgroup so far, thus the 16 remaining pregnancies are still ongoing (n = 2) or have resulted in live-birth (n = 14).

The mean age of our patients diagnosed with pelvic adhesions was 36.00 (±1.62) years old. The number of previous failed IVF attempts ranged from 3 to 6 with an average of 4.27 (±0.86) previous failed ICSI attempts. The years of infertility the patients reported ranged from 6 to 9, with an average of 7.14 (±0.76) years. Eight out of 22 patients presented with a positive hCG test. Seven out of 22 patients achieved a clinical pregnancy following the first ICSI cycle. No statistically significant difference was observed between the pregnant and the non-pregnant group (Supplementary Table 3). Only one miscarriage has been reported while the 6 remaining pregnancies have resulted in live-births.

The mean age of our patients diagnosed with male factor infertility only was 36.22 (±1.42) years old. The number of previous failed IVF attempts ranged from 3 to 6 with an average of 4.19 (±0.57) previous failed ICSI attempts. The years of infertility the patients reported ranged from 5 to 9, with an average of 7.44 (±0.98) years. Nine out of 36 patients presented with a positive hCG test. Six out of 36 patients achieved a clinical pregnancy following the singular ICSI cycle. No statistically significant difference was observed between the pregnant and the non-pregnant group for any of the parameters studied (Supplementary Table 4). No miscarriage has been reported so far thus the 6 remaining pregnancies are still ongoing (n = 1) or have resulted in live-birth (n = 5).

## Discussion

With the exception of severe cases of azoospermia, and oligoasthenoteratozoospermia, male factor infertility is largely regarded as a good prognosis regarding IVF success, second only to tubal factor infertility^13^. Since the majority of couples undergoing IVF- irrespectively of age and etiology -achieve a pregnancy within the first 3 cycles^14^, one can extrapolate that couples diagnosed with male factor infertility coupled by a reproductive history entailing more than 3 previous IVF cycles, should potentially explore further fertility investigation instead of attributing recurrent failed attempts strictly to the standing infertility etiology of male factor.

This study set out to test the theory that perhaps an underlying factor remaining unidentified could be contributing to futile IVF attempts and subsequent overuse in cases of a reproductive history of at least 3 failed IVF attempts and a good prognosis of mild male factor infertility. In an effort to identify the possibility of an underlying undiagnosed factor, design of the study included performing laparoscopy as the end-point in infertility investigation. According to our results for couples with mild male factor infertility and at least 3 previous failed IVF attempts, laparoscopy may enable underlying endometriosis correction in 42.57% (43/101). An additional 21.78% (22/101) of patients benefited from laparoscopy by correcting pelvic adhesions. Prevalence of endometriosis in this specific population is concordant with previous literature on patients diagnosed with unexplained infertility^15,16^.

Out of 169 couples that were eligible to participate in the present study, 101 opted for laparoscopic investigation, whereas the remaining 68 opted to not be submitted to laparoscopic investigation. The women that opted to not be submitted to laparoscopy, presented with a statistically significant lower number of years of infertility. This may have served as the critical point in their decision. Patients submitted to laparoscopy, regardless of the diagnosis, presented with a statistically significant higher clinical pregnancy and ongoing pregnancy/live birth rates. This is attributed to the fact that 64.35% of our patients were diagnosed and treated for a condition that was further hindering their fertility potential.

A marginally non statistically significant improvement in blastocyst quality was observed in the endometriosis group when compared to the male factor infertility only group. No difference was reported regarding top-quality blastocysts. The three groups did not present with any other statistically significant differences. This is in partial agreement with previous literature^17^. A possible reason behind this, is that Shahine and colleagues evaluated strictly the percentage of good quality blastocysts, whereas in the present study the difference originates from the poor-quality blastocysts. It may be possible that endometriosis correction does not affect the number of top-quality blastocysts, but decreases the number of poor-quality blastocysts.

This result is interestingly reflected in clinical pregnancy rates where couples following endometriosis correction presented with a statistically significant higher rate (39.53% vs 16.67%). Couples with pelvic adhesions correction revealed a similar trend (31.82% vs 16.67%), albeit not reaching the traditional threshold of statistical significance. Nevertheless, this may translate to a different conclusion depending on the sample size. It should be noted that the relatively small sample of the study is purely attributed to the strict inclusion and exclusion criteria. The increase in clinical pregnancy rate, following endometriosis correction, is supported by standing literature^18,19^. Similar clinical pregnancy rate improvements have been observed with respect to natural conception following corrective laparoscopy in patients with more than 3 failed IVF attempts and unexplained infertility^16,20^.

The aforementioned small sample size despite being attributed to the strict inclusion criteria presents as a limitation for the present study. Albeit results are encouraging, the fact that the study was conducted in a single center, highlights the lack of external validation. Single center studies conform to a single standard operating protocol that may not be shared by other centers. Moreover, the fact that patient group assigning with regards to undergoing laparoscopic investigation or not, was performed according to patient’s choice, rather than a computer-based randomization algorithm presents another limitation for the present study.

It is soundly hypothesized that the presence of undiagnosed pelvic pathologies, namely endometriosis, could adversely affect reproductive potential. For couples diagnosed with unexplained infertility or with male factor infertility, resulting to multiple implantation failures, it may be of added benefit to investigate the possibility of undiagnosed pathologies regarding the female partner. In most of the cases, basic infertility investigation, including clinical examination and TVUS, may offer substantial diagnostic information in respect to the possible pelvic pathologies compromising reproductive potential. TVUS may stand as the first-line diagnostic tool^21^. Nonetheless, a number of pelvic pathologies, such as superficial or deep endometriotic spots on the peritoneum or filmy adhesions on the ovaries, may not be detected via TVUS or/and clinical examination^16,20,22^. Interestingly, in four cases presented herein, it was laparoscopic investigation that revealed the existence of undiagnosed microscopic deep ovarian endometriosis lesions which were not detected via preoperative TVUS. It is well documented that TVUS is characterised by high sensitivity and specificity regarding ovarian endometriosis diagnosis^23^. However, in some cases endometriotic implants (superficial or deep) might be so small and thus not easily detected by standard TVUS^24,25^. Significant efforts towards improving TVUS markers are being made^25,26^. In light of the above, should diagnosis of unexplained infertility or male factor infertility cases presenting with repeated implantation failure, alarm clinicians? Data presented herein indicate that, in such cases, laparoscopic investigation might be an efficient diagnostic and therapeutic solution towards overcoming implantation failure and subsequent infertility.

Evidence from current studies suggest that endometriosis is a complex disorder, influenced by genetic and epigenetic mechanisms. Immune cells, adhesion molecules, extracellular matrix metalloproteinase and pro-inflammatory cytokines alter peritoneal microenvironment, enabling differentiation, adhesion, proliferation and survival of ectopic endometrial cells, resulting to impeding oogenesis, fertilization and implantation^27^.

Endometriosis presents with a prevalence of 6–10% in women of reproductive age, while a 20% of women with endometriosis present with deep infiltrating endometriosis (DIE)^28,29^. DIE is defined as the sub-peritoneal invasion by endometriotic lesions that exceed 5 mm in depth^30^. Intestinal involvement is present in 8–12% of these patients and colorectal implants are present in 90% of intestinal involvement^29^. Patients with DIE and colorectal implants require surgical treatment. This is performed via laparoscopy, employing a mesenteric nerve-sparring and vascular sparring approach in order to preserve mesentery arteries, and surrounding autonomic nerve fibers that reduces bowel denervation^28^. Surgical treatment of DIE, especially employing full excision of endometriotic lesions, may enable the restoration of normal pelvic anatomy and its function^31^. It should be mentioned that even following laparoscopic excision of endometriotic lesions, women with previous DIE, may present with significantly higher pregnancy complications, for example hypertensive disorders, placenta previa and lower gestational weight^32^.

The authors purposefully refrain from bold statements regarding a change in practice. The authors underline that laparoscopy was employed in a special cohort of patients that presented with mild male factor infertility and at least 3 failed IVF attempts. Male factor infertility, when employing ICSI, is not associated with further failed IVF attempts^33^ thus the average of more than 4 previous failures may not be justified solely on the grounds of mild male factor infertility etiology. Laparoscopy guidelines in the reproductive treatment context have been thoroughly presented even though controversy remains-mainly attributed to its invasive nature^34–36^. These are aspects that merit serious consideration regarding patient management. The patients’ desire and tolerance regarding possible options following numerous previous failed IVF attempts play a pivotal role. Recurrent failed IVF attempts may be associated with introducing various options for the couples including gamete donation^9^. Most of these patients would normally proceed with a consultation regarding gamete or embryo donation, a challenging option that may not always be welcomed by the patients. It should be mentioned that the guidelines that report on optimal management of male factor infertility are referring mainly on the diagnostic methods employed, or the pharmaceutical regime that should be administered. Only NICE guidelines ^7^refer to considering IVF for mild male factor, following 24 months of aiming to achieve a natural conception. The development of guidelines regarding management of mild male factor infertility and IVF is of great importance, since its management is hitherto considered empirical, relying on the success of the insemination method of choice being IVF or ICSI.

Development of a novel biomarker should be considered as a necessity to properly address justification of laparoscopic investigation in light of its invasive nature. The invasive nature of laparoscopy may have been a determining factor in couples opting against laparoscopic investigation without any indication. Albeit our results highlight the efficiency of laparoscopic investigation-following more than 3 previous failed IVF attempts, excluding any severe infertility factor- more than 35% of our patients were submitted to this procedure failing to result in a successful diagnosis or to an enhanced pregnancy rate.

## Conclusion

Our findings suggest that patients, who fall under the category of strict inclusion and exclusion criteria, could benefit from laparoscopic investigation and correction. These patients present with a reproductive history of more than 3 failed IVF attempts attributed to an infertility etiology of mild male factor with no further identification of any female factor etiology. Thus, perhaps it may be time to reconsider the place of laparoscopy in completing the infertility investigation for such cases. This may be particularly true for couples where the etiology may not stand to adequately justify futile IVF attempts. The important role of patients’ will especially in cases where laparoscopy may be viewed as the end-point of fertility treatment should be respectfully weighed in the equation regarding optimal fertility treatment.

## Materials and Methods

Study population of the current prospective cohort study included couples, initially diagnosed with male infertility, attributed to mild oligozoospermia (>5–10 × 10^6^/ml) coupled with at least 3 unsuccessful ICSI cycles. The definition of an unsuccessful ICSI cycle for this study refers to cycles that followed by an embryo transfer they failed to lead to a positive hCG test. The enrollment of participants was conducted from February 2015 to July 2018. The last ICSI cycles were performed on February 2019. All ICSI cycles were performed at least 7 months following laparoscopy. All embryo transfers were performed including blastocyst stage embryos. Blastocyst quality was assessed according to Gardner’s grading system^37^. The grading was performed without the embryologists being aware of the patients’ participation in the study.

Standard fertility investigation was performed, entailing semen analysis, and evaluation of ovarian function through assessment of hormonal levels including follicle stimulating hormone (FSH),and luteinizing hormone (LH), recorded on day 3 of the menstrual cycle, while progesterone levels were assessed on day 21 of the menstrual cycle, prior to initiation of stimulation. Evaluation of these levels provided data regarding the ovarian function of the patient. Estradiol (E_2_) levels were assessed on the day of ovulation trigger, aiming to provide data regarding adequacy of response to stimulation, and this was coupled by ultrasound screening. Semen analysis was performed according to World Health Organization (WHO) 2010 criteria^38^. Patients diagnosed with asthenozoospermia, teratozoospermia, azoospermia, severe oligozoosperma (<5 × 10^6^/ml) and abnormal DNA fragmentation index were excluded on the grounds of limiting confounding factors that could present as accountable in cases of aneuploidy and recurrent pregnancy loss^39–41^. In order to confirm diagnosis of oligozoospermia two different semen analyses were performed with a 75-day interval. All participants were normo-ovulatory and presented with patent fallopian tubes, according to the hysterosalpingography results. These couples initially reported with no additional female-related infertility diagnosis, regarding the female partner’s perspective. The inclusion criteria regarding the women were normal karyotypes, FSH < 12 mIU/mL, LH < 12 mIU/mL measured on the day 3 of menstrual cycle, normal anatomy of the uterine cavity and functional fallopian tubes as confirmed by hysterosalpingography. With respect to the inclusion and exclusion criteria for the study, all women were younger than 40 years old, with no other infertility aetiology following basic infertility investigation. Women with current or previous cancer diagnosis, auto-immune, genetic or reproductive disorders were excluded from the present study. Couples presenting with a reproductive history of miscarriage due to genetic abnormalities or requesting PGT-M cycle were similarly excluded from the present study.

The women included in our study, underwent laparoscopy as a last resort, aiming to identify and correct hitherto undiagnosed endometriosis. The presence of endometriotic lesions and adhesions and the stage of the disorder were determined according to the revised American Fertility Society (rAFS) classification of the ASRM. Laparoscopies were performed under general anesthesia. Following corrective laparoscopy, patients proceeded with a single ICSI cycle. Abiding by the national legislation and in order to avoid bias due to discrepancies regarding the number of embryos transferred, two blastocysts were transferred for each cycle. The quality of the embryos transferred was cumulatively considered as top-quality in the case that both transferred blastocysts were graded as 4–6AA, and classified as poor quality in the case that both transferred blastocysts were graded as either BC, CB, or CC. Regarding all other cases including combinations of blastocysts of intermediate grading, or including one blastocyst of top and one of poor quality, they were described as of moderate quality. Regarding previous cycles only women who presented with strictly top or poor quality embryos transferred in all cycles were classified in the respective categories. All other cases were classified as moderate.

Our primary outcome measure was clinical pregnancy rate, while the secondary outcome measures were live-birth/ongoing pregnancy rate, and positive hCG rate. Clinical pregnancy was confirmed by detection of an intrauterine gestational sack and fetal heartbeat detectable via ultrasound monitoring 6 to 7 weeks following the last menstrual cycle. Live-birth and ongoing pregnancies are regarded as a single result and reported on collectively. This is due to the fact that understandably a number of women recruited at the later stages of the present study did not give birth within the completion time-frame of the study. The Center for Human Reproduction’s Ethics Board approved the study protocol (292/09-12-2014) in accordance to the Helsinki declaration and following patients’ written consent, operative and corrective laparoscopy was performed. Women who opted against laparoscopy also signed the informed consent form regarding their participation to the study. The patients that were classified as candidates for recruitment were provided adequate time to read and understand the consent form and present with questions to the treating physician. The consent form informed the patients-employing layman’s terms-regarding the procedure that laparoscopic surgery entails and any possible adverse effects, along with the fact that the respective IVF treatment pursued by the patients would not be subjected to any modification regardless of their decision to participate or not in the study. Moreover, the patients were informed that the participation and subsequent data provided from the cycles would be extracted and analyzed in an anonymous, decrypted method. The patients were invited by the treating physician to decide-in their own time-on the following options: to undergo laparoscopy and participate in the study, to participate in the study without performing laparoscopy and to not participate in the study.

### Laparoscopic procedure

Laparoscopic procedure was performed according to the ESHRE 2013^42^ and the NICE 2017 Guidelines^43^. The classification system of endometriosis stage was according to ASRM 1997^44^. This combines the evaluation of ectopic lesions in ovaries and peritoneum regarding the abnormal morphology, number, size or position of ectopic lesions, resulting to a total score. Based on the provided score, diagnosed endometriosis is classified as Stage I, II, III, and IV. During laparoscopy, surgical ablation or resection of endometriotic lesions plus adhesiolysis was performed for all women with stage I/II endometriosis. In cases of stage III/IV endometriosis with deep peritoneal endometriotic lesions, deep ovarian endometriosis, cul-de-sac obliteration and dense ovarian and tubal adhesions, adhesiolysis was performed and excision of the lesions with the employment of CO_2_ laser evaporation. In women with endometrioma, cystectomy with excision of the endometrioma capsule was performed selectively always considering the women’s ovarian reserve. Tubal flushing was performed for all laparoscopic procedures.

### Statistical analysis

All data analyses were performed using the R programming language. Normality of the distribution was evaluated with the Shapiro-Wilks test. Students’ t-test or Mann-Whitney U was employed to investigate statistically significant differences between groups according to the distribution of data. The evaluation of statistically significant differences between subgroups was performed employing One-Way Anova or Kruskal-Walis according to the distribution of data. Since no statistically significant difference was observed, in the continuous variables of the subgroups, no post-hoc test was employed. Positive hCG test and clinical pregnancy rates were compared employing odds ratio.

## Supplementary information

Supplementary tables.
