# Supplementary material for: Couples with mild male factor infertility and at least 3 failed previous IVF attempts may benefit from laparoscopic investigation regarding assisted reproduction outcome
Source: Sci Rep. 2020 Feb 11;10:2350. doi: 10.1038/s41598-020-59170-5 (PMC7012822; doi:10.1038/s41598-020-59170-5)
Supplement: Supplementary file 1 — Supplementary tables. [file 41598_2020_59170_MOESM1_ESM.pdf]

"Couples with mild male factor infertility and at least 3 failed previous IVF attempts may benefit from laparoscopic investigation regarding assisted reproduction outcome "

Agni Pantou[a*], Konstantinos Sfakianoudis[a*], Evangelos Maziotis[b], Polina Giannelou[a,b], Sokratis Grigoriadis[b], Petroula Tsioulou[b], Georgia Kokkali[a], Michael Koutsilieris[b], Konstantinos Pantos[a*], Mara Simopoulou[b,c*]

[a] Centre for Human Reproduction, Genesis Athens Clinic

[b] Department of Physiology, Medical School, National and Kapodistrian University of Athens

[c] Assisted Conception Unit, 2nd Department of Obstetrics and Gynaecology, Aretaieion Hospital, Medical School, National and Kapodistrian University of Athens

*Please consider joint first and last authorship

Corresponding Author

Mara Simopoulou, Assistant Professor of Physiology, Senior Clinical Embryologist/Geneticist, Department of Physiology-Department of Obstetrics and Gynaecology, Medical School, National and Kapodistrian University of Athens, 75, Mikras Asias 11527, Athens, Greece, Tel/Fax: +302107462592, +302107462571, E-mail: marasimopoulou@hotmail.com

Supplementary Tables

Supplementary Table 1: Description of different pathologies diagnosed and treated during laparoscopic surgery

LAPAROSCOPIC FINDINGS	CLASSIFICATION OF ENDOMETRIOSIS	N (%)	DIAGNOSIS FOLLOWING LAPAROSCOPIC INVESTIGATION
Normal pelvic anatomy without endometriosis or adhesions or any other pathology	No pathologies	36	Male Factor Only
Superficial endometriotic spots (1-3cm) on peritoneum	Stage I	9	Endometriosis (N= 43)
Superficial endometriotic spots (1-3cm) on peritoneum and superficial endometriotic spots (<1cm) and filmy adhesions on right ovary	Stage I	5	
Deep endometriotic spots (>3 cm) on peritoneum	Stage II	11	
Deep endometriotic spots (>3cm) on peritoneum and superficial endometriotic spots (<1cm)	Stage II	8	
Deep endometriotic spots (>3cm) on peritoneum and superficial endometriotic spots (<1cm) and filmy adhesions on right ovary and superficial endometriotic spots (<1cm) on left ovary	Stage II	6	
Deep endometriotic spots (>3 cm) on peritoneum and deep endometriotic spots (1–3 cm) on left ovary and partial cul-de-sac obliteration	Stage III	3	
Superficial endometriotic spots (>3cm) on peritoneum, filmy adhesions on right fallopian tube/right ovary, deep endometriotic spots (<1 cm) dense adhesions on left ovary and dense adhesions on left tube	Stage III	1	
Periadnixal and pelvic adhesions without endometriosis	Only Adhesions	22	Adhesions

Supplementary Table 2: Descriptive statistics of the endometriosis group

	Pregnant (N=17)	Not-Pregnant (N=26)
**Age**	36.76±0.94	36.35±1.41
**Years of Infertility**	7.00±0.97	7.35±0.87
**Previous IVF attempts**	4.76±0.73	4.27±0.81
**FSH (U/ml)**	4.06±0.61	4.44±0.64
**LH (U/ml)**	3.65±0.87	3.85±0.86
**Estradiol (pg/ml)**	2937.59±85.84	2856.15±177.25
**Progesterone (ng/ml)**	11.96±1.49	13.09±1.82
**CA-125 (U/ml)**	18.33±2.77	18.21±2.81
**Oocytes Retrieved**	10.88±2.3	11.58±2.08
**MII oocytes**	9.76±2.26	9.58±2.19
**Fertilized oocytes**	8.71±1.81	8.46±1.82

**Blastocysts**	5.12±1.02	4.92±1.11

Supplementary Table 3: Descriptive statistics of the pelvic adhesions group

	**Pregnant (N=7)**	**Not-Pregnant (N=15)**
**Age**	36.00±1.69	36.00±1.59
**Years of Infertility**	7.43±0.49	7.00±0.82
**Previous IVF attempts**	4.00±0.53	4.40±0.95
**FSH (U/ml)**	4.11±0.60	4.56±0.72
**LH (U/ml)**	4.05±0.73	3.57±1.02
**Estradiol (pg/ml)**	2844.00±130.52	2823.8±145.93
**Progesterone (ng/ml)**	12.75±1.40	12.37±2.28
**CA-125 (U/ml)**	17.61±3.66	18.22±2.27
**Oocytes Retrieved**	10.14±1.12	10.60±1.96
**MII oocytes**	9.43±1.18	9.73±1.77
**Fertilized oocytes**	8.29±1.03	8.47±1.31
**Blastocysts**	4.57±0.49	4.93±0.93

Supplementary Table 4: Descriptive statistics of the male factor infertility only group

	**Pregnant (N=6)**	**Not-Pregnant (N=30)**
**Age**	37.00±1.29	36.07±1.39
**Years of Infertility**	7.17±1.34	7.50±0.89
**Previous IVF attempts**	4.17±0.37	4.20±0.6
**FSH (U/ml)**	4.58±0.88	4.20±0.74
**LH (U/ml)**	4.03±0.79	3.72±0.82
**Estradiol (pg/ml)**	2840.17±78.72	2830.03±143.03
**Progesterone (ng/ml)**	13.29±1.81	13.34±1.17
**CA-125 (U/ml)**	16.11±3.99	17.94±3.00
**Oocytes Retrieved**	10.67±1.97	10.43±1.50
**MII oocytes**	8.50±0.96	8.93±1.34
**Fertilized oocytes**	7.50±0.96	7.67±1.11
**Blastocysts**	4.33±0.75	4.53±0.76
